# Confounding Effects of Phase Delays on Causality Estimation

**DOI:** 10.1371/journal.pone.0053588

**Published:** 2013-01-21

**Authors:** Vasily A. Vakorin, Bratislav Mišić, Olga Krakovska, Gleb Bezgin, Anthony R. McIntosh

**Affiliations:** 1 Rotman Research Institute, Baycrest Centre, Toronto, Ontario, Canada; 2 Department of Psychology, University of Toronto, Toronto, Ontario, Canada; 3 Department of Chemistry, York University, Toronto, Ontario, Canada; Cuban Neuroscience Center, Cuba

## Abstract

Linear and non-linear techniques for inferring causal relations between the brain signals representing the underlying neuronal systems have become a powerful tool to extract the connectivity patterns in the brain. Typically these tools employ the idea of Granger causality, which is ultimately based on the temporal precedence between the signals. At the same time, phase synchronization between coupled neural ensembles is considered a mechanism implemented in the brain to integrate relevant neuronal ensembles to perform a cognitive or perceptual task. Phase synchronization can be studied by analyzing the effects of phase-locking between the brain signals. However, we should expect that there is no one-to-one mapping between the observed phase lag and the time precedence as specified by physically interacting systems. Specifically, phase lag observed between two signals may interfere with inferring causal relations. This could be of critical importance for the coupled non-linear oscillating systems, with possible time delays in coupling, when classical linear cross-spectrum strategies for solving phase ambiguity are not efficient. To demonstrate this, we used a prototypical model of coupled non-linear systems, and compared three typical pipelines of inferring Granger causality, as established in the literature. Specifically, we compared the performance of the spectral and information-theoretic Granger pipelines as well as standard Granger causality in their relations to the observed phase differences for frequencies at which the signals become synchronized to each other. We found that an information-theoretic approach, which takes into account different time lags between the past of one signal and the future of another signal, was the most robust to phase effects.

## Introduction

Rhythmic activity generated by individual neurons or by interactions between neurons is a widely observed phenomenon in the brain [1]. Firing patterns of a group of neurons may become synchronized [2]. Synchronized activity of neural ensembles may lead to macroscopic oscillations [3], which can be detected with measurements of local field potentials (LFP), electroencephalographic (EEG), or magnetoencephalographic (MEG) recordings. From a mathematical point of view, the underlying neural ensembles can be represented by single oscillators [4]. In turn, different neural ensembles can be coupled with long-range connections, forming a large-scale network of coupled oscillators. Numerous studies have shown that cognitive function can be explained in terms of synchronous dynamics of large neuronal ensembles coupled within and across subsystems [5]. Encouraging results were obtained in modeling the resting state network dynamics wherein time delays play a crucial role in the generation of realistic fluctuations in brain signals [6], [7].

Phase synchronization between coupled neural ensembles is considered a mechanism implemented in the brain to integrate relevant neuronal populations at a given moment to construct task-related functional networks [8]. Mathematical implementation of phase synchronization is driven by an idea that the existence of relations between phases of coupled systems does not necessarily imply the correlation between their amplitudes [9]. A number of techniques have been employed to study synchronization between oscillating brain signals (for a review see [10] or [11]). Among others, analysis of phase-locking is a popular approach, wherein the robustness of the phase differences (across trials or time points) between pairs of sensors/regions is quantified in a statistical sense [12], [13]. Strong phase-locking effects can often be observed in brain signals, for example, as a reaction to performing a cognitive or perceptual task [8], [14].

Another framework to gain insight into the mechanisms underlying functional networks is to uncover the directionality of interactions between coupled systems. The notion of Granger causality was introduced based on an idea of asymmetry in signals' ability to predict each other [15]. Under this framework, a process 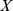 is considered a cause of another process 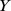, if the incorporation of the knowledge about the past of 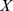 significantly improves the prediction of the future of 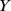, compared to the prediction that is based only on the knowledge about the past of 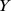. The asymmetry in enhancement of predictive power between signals would indicate the directionality of coupling between the presumably coupled systems underlying the observed signals (see [16] for a review).

The original concept of Granger causality was formulated in terms of autoregressive processes. Excluding a translation from a bivariate version to multivariate models, two extensions of Granger causality are proposed in the literature. The first one is a spectral version of Granger causality that is based on the Fourier transform of autoregressive models [17]. In such a case, asymmetry in predictive power is frequency specific, providing more information on the strength of mutual interdependencies between brain waves for a given range of frequencies [18]. Such a methodological perspective found a number of applications in the analysis of neurophysiological signals, including LFP,EEG, MEG, and functional magnetic resonance imaging (fMRI) [19]–[21].

Analytic tools provided by information theory are a way of constructing non-linear versions of Granger causality in the time domain. Under the information-theoretic approach, we do not need to specify *a priori* a model of signals and their interactions. Instead, the transfer of information from the past and present of one process to the future of another process can be quantified in terms of individual and joint entropies, which measures the amount of uncertainty contained in the observed signals. The transfer of information is essentially a conditional mutual information [22]. Another statistic called transfer entropy [23] is shown, under certain conditions, to be equivalent to the measure of conditional mutual information [24]. The attractive “model-free” property of information-based statistics has lead to numerous applications in neuroimaging and neurophysiological studies. Specifically, transfer entropy has been applied in both EEG [25]–[27] and MEG data [28]–[30], as well in fMRI [31].

It should be emphasized that the notion of Granger causality is based on the idea of temporal precedence where a cause precedes its consequences. In the case of distinct harmonic components, time delay, in general, cannot be converted into phase delay without ambiguity due to shifting a wave backward or forward a full cycle (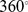). In the case of a linear transfer function, the slope of the phase over a range of frequencies, which is the group delay, can be used to overcome the phase ambiguity that exists at a specific frequency [32], [33]. However, the situation is different in the case of a coupled non-linear system with a possible time delay in coupling.

In a network, wherein each coupling between two nodes can be characterized by its own connection strength, directionality, and time delay in coupling, the true temporal precedence between two signals may materialize as either phase delays or phase advances at specific frequencies. Furthermore, one can hypothesize that the effects related to what is observed as a phase delay may counteract the effects related to the temporal precedence. This might be of special relevance to inferring the directionality of coupling based on spectral decomposition of the original signals. In this study, we controll the parameters of coupling to show that, given the same directionality of coupling, as specified by the underlying model, we can observe either phase delay or phase lead between the driver and the response. In turn, this phase lag affects the estimation of Granger statistics. Three measures of causality are compared in this study, namely the standard and spectral Granger causality as well as its information-theoretic version, in their relations to the observed phase differences for frequencies at which the signals become phase-locked to each other. At the same time, the phase-locking index is used to assess phase relationships between the signals.

## Materials and Methods

### Granger causality

Suppose that the dynamics of two processes 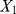 and 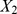 are described by an autoregressive model:
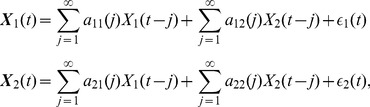
(1)which, in case of finite time series, is reduced to a model based on 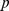 lagged observations:
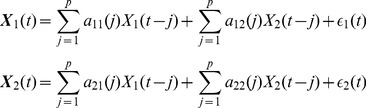
(2)where 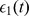 and 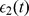 are the prediction errors for each time time series. According to [15], if the variance of 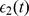 is reduced by including the terms 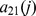 in the second equation of (2), compared to keeping 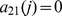 for all 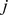, then 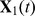 is thought to be causing 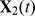.

Formally, an enhancement of predictive power can be quantified as follows. Suppose that 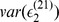 is the variance of noise 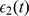 derived from a model with 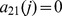 for all 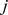. Also, let 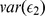 be the variance of the same residuals derived from the full model (2). The Granger causality 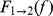 from 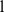 to 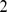 is then defined as
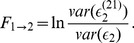
(3)In a similar way, we can define 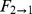, which quantifies causality from 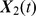 to 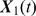. The asymmetry in two measures, 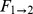 and 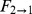, may indicate the directionality of coupling between 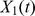 and 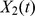:
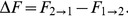
(4)Thus, if 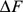 is positive, the directionality of coupling is thought to be as 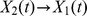, and *vice versa*.

### Spectral Granger causality

In this section, we describe a measure of spectral Granger causality [17]. Suppose that the dynamics of two processes 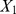 and 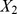 are described by an autoregressive model as specified in (2) with the noise covariance matrix 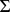. An interpretation of Granger causality in the frequency domain can be derived through the Fourier transformation of the autoregressive model in the time domain:
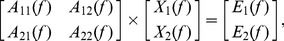
(5)where 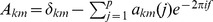, 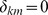, if 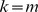, and 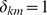, if 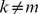. In terms of the transfer functions 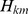, the model (5) reads as
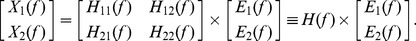
(6)The spectral matrix 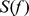 is defined as 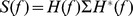, where 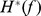 is the conjugate transpose of 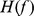. The spectral Granger causality 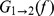 from 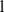 to 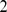 is then defined as
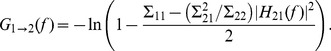
(7)In a similar way, the spectral Granger causality 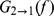 from 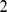 to 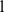 can be defined. The difference between 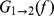 and 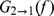 may indicate the directionality of coupling between 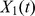 and 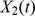 at a specific frequency:
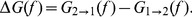
(8)Thus, if 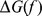 is positive, the directionality of coupling at frequency 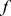 is reconstructed as 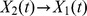, and *vice versa*.

### Information-theoretic causality

Now we consider a non-linear causal statistic that works in the time domain. Suppose that we observe two processes, 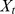 and 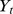, and our goal is to reconstruct the directionality of couping between them, if any. Following [22] and [24], a statistic can be designed using a combination of information-theoretic tools and concepts from non-linear dynamics. In contrast to (1), using information theory, there is no need to assume that there exists a specific model describing the processes 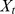 and 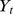. However, we assume that 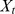 and 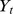 are realizations of two non-linear dynamic models, underlying the observed signals.

In this case, we need to reconstruct, from a time series of observations, the dynamics in the multi-dimensional state space of the underlying model. This can be done with time delay embedding
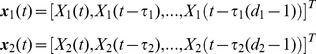
(9)where 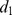 and 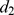 are embedding dimensions, and 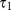 and 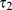 are embedding delays measured in multiples of the sampling interval. Thus, the time series 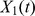 and 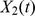 are converted to a sequence of vectors in an 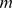-dimensional space.

The coupling directed from 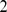 to 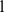, 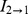, can be quantified by estimating the extra amount of information about the future values 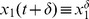 of 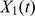, contained in the delay vector 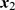 provided that the knowledge about the past of 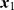 is excluded. This extra information can be quantified as the conditional mutual information 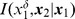 between 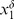 and 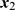 given 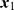. It can be estimated in terms of individual 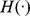 and joint entropies 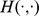 and 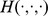 of the processes 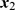, 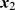, and 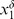 as follows:
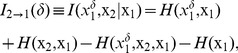
(10)where the time lag 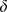 between the future and the past of a signal is typically measured in multiples of the sampling interval. It can be shown that under certain conditions, 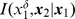 is equivalent to the measure called transfer entropy [23], [24].

In a similar way, we can define the coupling from 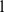 to 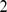, 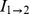, as the mutual information between the future of 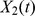 and the past of 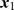, *e.g.* between 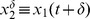 and 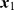, given that we exclude the knowledge about the past of 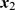:
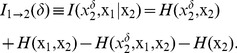
(11)Similar to (8), the difference in two measures, 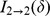 and 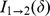, may indicate the directionality of coupling between 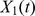 and 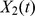 at a specific time lag 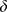:
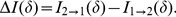
(12)Thus, if 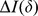 is positive, the directionality of coupling at the time lag 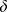 is inferred as 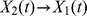, and 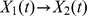, if negative.

### Phase locking and phase delay

The phase-locking index (PLI) is known in the literature under different names such as mean phase coherence [12] or phase synchronization index [13]. The PLI is able to quantify phase synchronization between signals in a statistical sense, and emerged from studying coupled non-linear systems [9]. In turn, phase synchronization is based on an idea that the existence of relations between phases of coupled systems does not necessarily imply the correlation between their amplitudes.

Suppose that there are 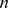 realizations of two processes 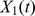 and 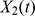. Phase-locking between channels across realizations can be computed using the concept of frequency-specific phase difference between the signals. Specifically, the cross spectrum 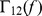 as a function of frequency 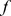 between two signals 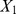 and 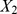 has the form of
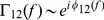
(13)where 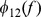 is the cross phase spectrum. The function 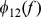 represents the phase shift between the two signals at a specific frequency 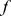.

With 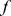 fixed, 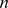 realizations of 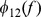 can be described as a distribution of the radius vectors of unit length in the complex space. The phase locking index 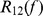 is computed as the length of the mean vector obtained by averaging the radius vectors 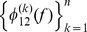 in the complex space across realizations. Specifically,
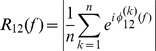
(14)


The phase locking index represents a frequency-specific measure to quantify the amount of phase synchrony inherent in two given signals. By design, the statistic 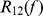 is limited between 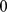 and 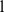. When the cross phase distribution is highly concentrated around its mean, the PLI is close to one. The PLI is close to zero for the uniformly distributed phase differences across realizations.

Together with the PLI, we can compute the mean phase delay 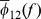 between two signals, averaged across the realizations. However, there is an ambiguity in cumulative phase shift between harmonic signals as, in general, it is unknown how many cycles the phase completed. In this study, we define the observed phase delay or phase lead in a such a way that the phase difference 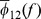 between 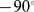 and 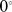 implies that the signal 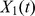 (response) is delayed with respect to 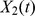 (driver) at frequency 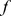.

### A model of coupled oscillators

To study the influence of phase locking and phase delay on causality estimation, a system of coupled Rössler oscillators is used. Such a model represents a relatively simple non-linear system able to generate self-sustained non-periodic oscillations. It should be noted that oscillatory behavior of the brain rhythms have been extensively studied as a plausible mechanism for neuronal communication [5], [8]. Under this context, the coupled Rössler oscillators can be viewed as a prototypical example of oscillatory networks. Coupled Rössler systems were used to study collective dynamics in oscillatory networks as a simple case of periodic systems perturbed by a noise that has a deterministic rather than stochastic nature [34].

Explicitly, the model reads
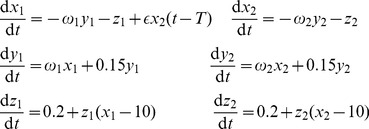
(15)where 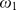 and 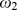 are the natural frequencies of the oscillators, 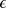 is the coupling strength, and 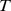 denotes the delay in coupling. Roughly speaking, each Rössler system represents an oscillatory trajectory in the 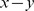 plane, with spike-like behavior in the 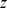 direction. Further analysis is based on an assumption that only the dynamics of the variables 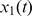 and 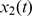 can be observed.

### Estimation

All cases considered in this study were based on the model (15) with 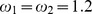 and the directionality of coupling 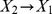. Numerical solutions of Eqs. (15) were obtained using the dde23 Matlab function (the Mathworks, Natick, MA) with a subsequent resampling of the time series with a fixed step 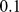 s. The dynamics were solved on the interval 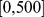
s, subsequently discarding the interval 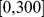
s to avoid transitory effects. Thus, each time series had 2000 data points. The time axis was then re-scaled as 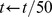, defining the new sampling interval of 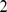 ms. Thus, the generated signals were designed to have the length of 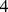 s with the sampling rate of 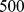 Hz, and represent oscillations approximately at 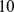 Hz. Then, the time series were normalized to have the mean of zero and variance of one. Gaussian noise (zero mean, variance of 0.3) was added to the signals.

Each case was characterized by a pair of the model parameters, the coupling strength 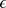 and the time delay in coupling 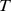. For a given pair of 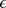 and 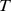, 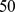 realizations of the model (15) were generated. For each realization, we generated a corresponding pair of surrogate time series, which are artificial data that mimic some properties of the original data. Surrogate data are constructed in a way such that some linear properties of the original signals remain unchanged, but causal relationships are destroyed. We generated surrogate signals according to a method designed to test pseudo-periodic data [35]. Thus, for each pair of 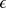 and 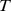, two ensembles of the original and surrogate time series were created, based on 50 realizations of the model of coupled oscillators.

For given 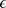 and 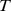, the phase-locking index and mean phase shift between harmonic components of the two signals were computed at the frequencies 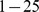 Hz. In addition, analyses of standard Granger causality, spectral Granger causality and transfer entropy were performed, separately for each realization, creating two ensembles of the test statistics for the original and surrogate data. By design, the measure 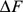 produced one value for each simulation. Depending on our purpose, we computed the measures 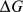 and 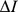 either as functions of frequency 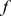 and the time lag 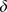 (three cases considered further), or as cumulative statistics, averaging across a range of 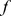 and 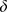, respectively. Bayesian information criterion [36] was used to determine an optimal order of the autoregressive model (2). The spectral statistic was estimated for frequencies 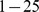 Hz. Transfer entropy was estimated for the time lags 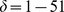 data points with the step of 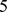 data points. The embedding delay was 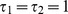 data point, and the embedding dimension was 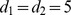. It should be noted that the dimension of the state space of systems with time delays is, in general, infinite. We used relatively large values for 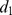 and 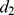, however, the results reported in this study were qualitatively robust with respect to a wide range of the embedding delay and dimension (not shown). The individual and joints entropies were estimated by computing the corresponding correlation integrals, as proposed by [37], and tested, with regards to inferring causal relations, using linear and non-linear models [25], [26], [38]. Cross-power spectral density of the time series was estimated using Welch's averaged, modified periodogram method of spectral estimation [39].

## Results

### Synthetic data

#### Three scenarios

**Figure 1:**
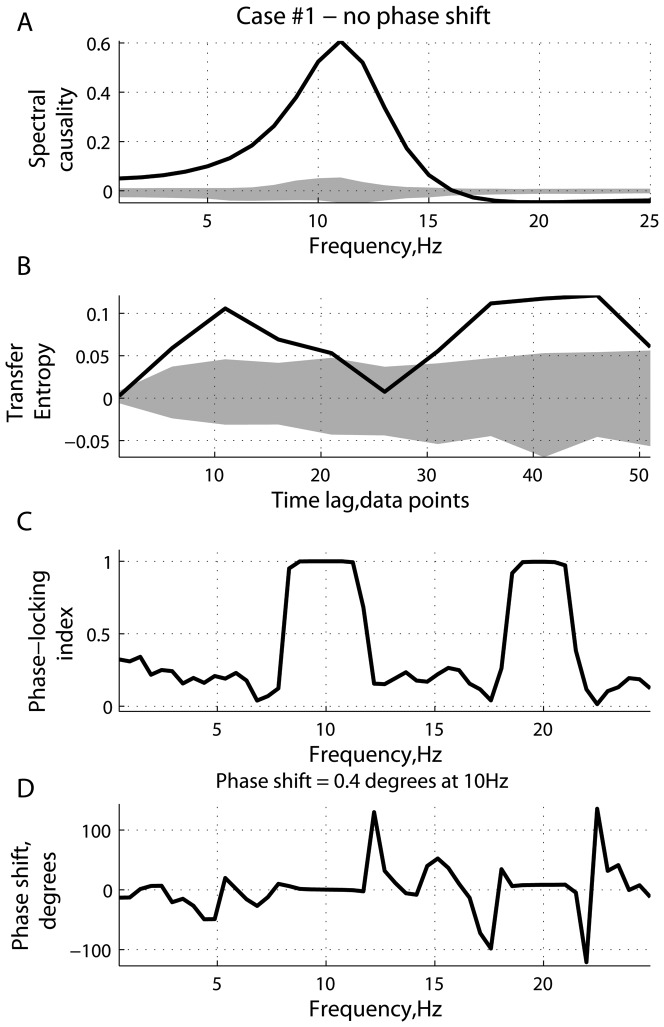
Scenario 1: causality and phase synchronization. Relations between the reconstructed causality and effects of phase-locking and phase differences in the case where there is no phase shift at the main frequency (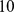 Hz): (A) measure of spectral Granger causality as a function of frequency; (B) transfer entropy as a function of the time lag 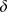 between the past of one signal and the future of the other; (C) phase-locking index and (D) phase shift as functions of frequency.

**Figure 2:**
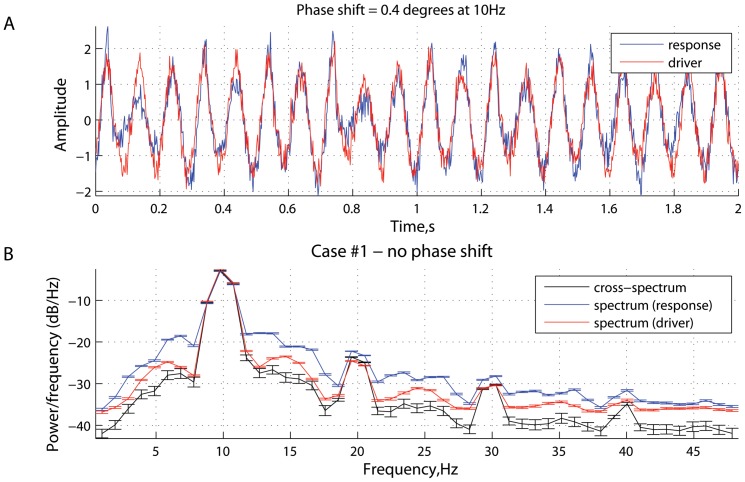
Scenario 1: time series and spectral power. Characteristics of the driver and response in the case where there is no phase delay (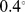) between them at 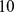 Hz (see Fig. 1): (A) simulated signals (two seconds of a randomly chosen realization); and (B) mean spectral density and cross power spectral density, averaged across realizations. The errorbars represent the standard error computed across realizations.

**Figure 3:**
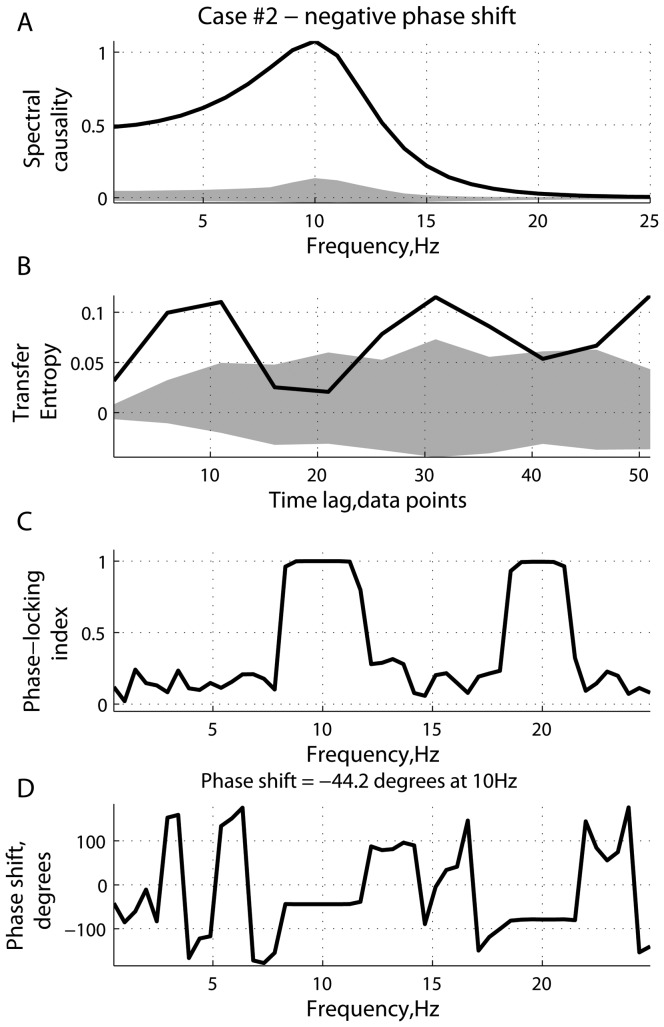
Scenario 2: causality and phase synchronization. Relations between the reconstructed causality and phase-related effects in the case where the phase shift between the driver and response is 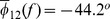: (A) measure of spectral Granger causality as a function of frequency; (B) transfer entropy as a function of the time lag 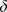; (C) phase-locking index and (D) phase shift as functions of frequency.

**Figure 4:**
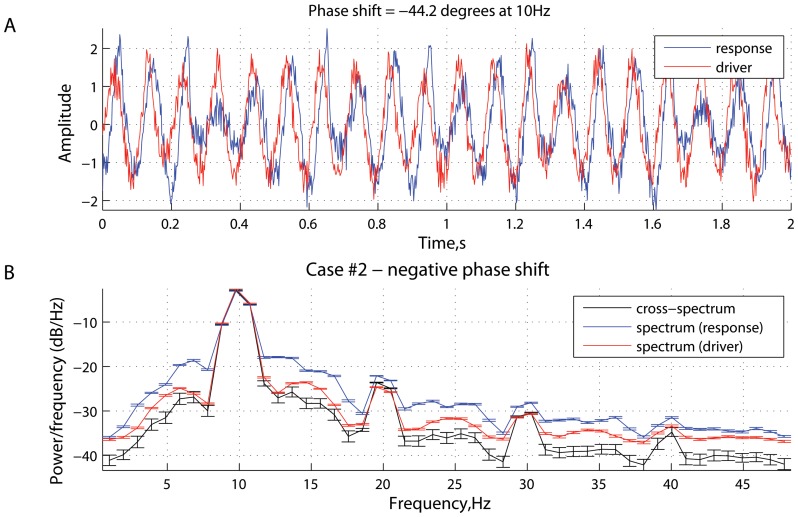
Scenario 2: time series and spectral power. Characteristics of the driver and response in the case of a negative phase difference (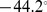) between them at 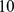 Hz (see Fig. 3): (A) simulated signals (two seconds of a randomly chosen realization); and (B) mean spectral density and cross power spectral density, averaged across realizations. The errorbars represent the standard error computed across realizations.

**Figure 5:**
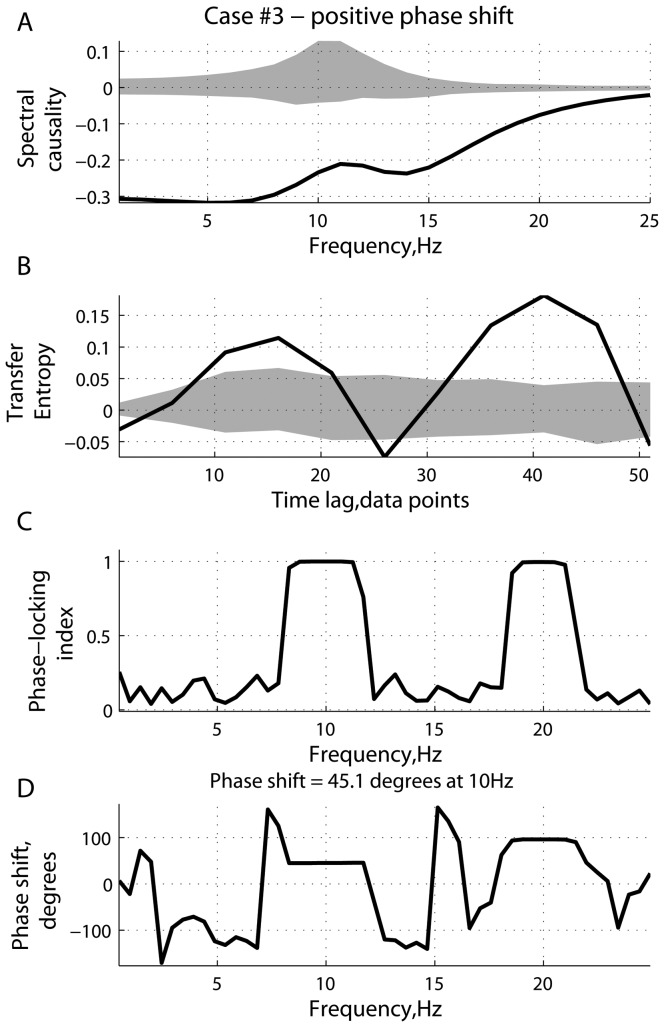
Scenario 3: causality and phase synchronization. Relations between the reconstructed causality and phase-related effects in the case of a positive phase difference (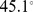) between them at 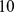 Hz: (A) measure of spectral Granger causality as a function of frequency; (B) transfer entropy as a function of the time lag 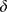; (C) phase-locking index and (D) phase shift as functions of frequency.

**Figure 6:**
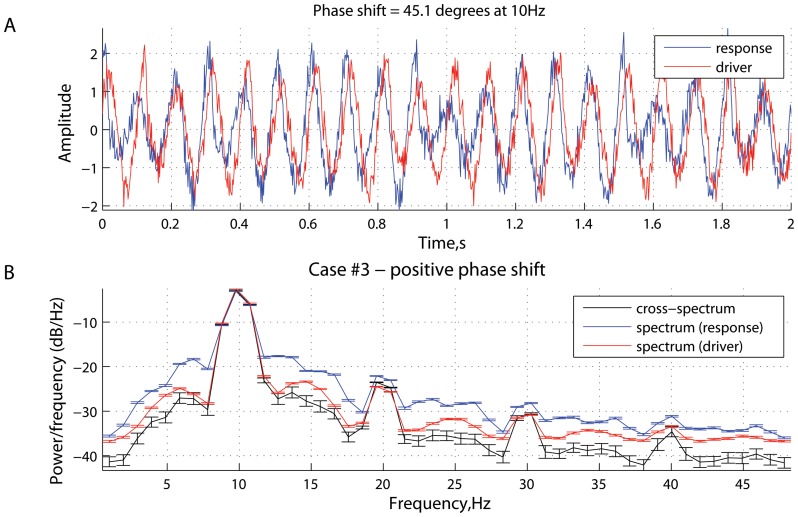
Scenario 3: time series and spectral power. Characteristics of the driver and response in the case of a positive phase difference (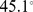) at 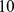 Hz (see Fig. 5): (A) simulated signals (two seconds of a randomly chosen realization); and (B) mean spectral density and cross power spectral density, averaged across realizations. The errorbars represent the standard error computed across realizations.

Figures 1, 2, 3, 4, 5, 6 represent three scenarios, showing an interplay between causality estimation and phase differences, with the parameters defining the system (15), as follows: 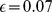 and 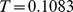 (Fig. 1 and 2); 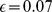 and 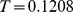 (Fig. 3 and 4); and 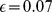 and 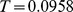 (Fig. 5 and Fig. 6). Specifically, Figures 1, 3, and 5 show: (a) spectral Granger causality 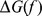, (c) phase locking index 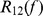 and (d) phase difference 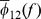 as functions of frequency 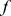, and (b) transfer entropy 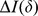 as a function of the time lag 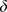. Figures 2,4, and 6 show the corresponding simulated signals (two seconds of randomly chosen realizations), and power spectra as well as cross-spectrum. As can be seen from panels (c), in all the cases, the signals become phase-locked at 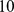 Hz. As a note here, 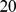 Hz represents a higher harmonic of the oscillations of 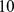 Hz. In all the panels, a solid line represents the mean of causal statistics under consideration, averaged across 50 realizations for the original data. The limits of the dark grey area are defined by the 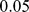
- and 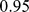-quantiles computed using the corresponding surrogate data.

In the scenario shown in Fig. 1 and 2, the parameters 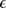 and 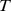 were chosen such that the phase difference at 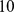 Hz was close to zero. In such a case, the measure of 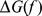 is positive for frequencies 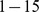 Hz, reaching a peak around 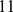 Hz (Fig. 1a). Positive values for 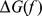 imply that the directionality of coupling is reconstructed like 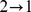. At the same time, the measure of transfer entropy was also positive for all time lags 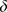, implying that the dominant transfer of information is going from system 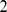 to system 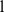.

In Fig. 3, the phase difference 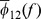 between signals 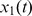 and 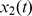 at 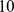 Hz is about 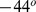, interpreted as the phase advance of the driving signal 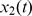 with respect to the responding 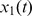. In this scenario, the time precedence, as specified by the modeled directionality of coupling concurs with the phase precedence, as detected from the phase-locking analysis (Fig. 4). In such a case, similar to Fig. 1, the measure of spectral Granger causality is positive, reaching a peak around 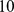 Hz. Positive values of 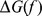 imply that the causal relations are reconstructed as 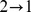. Note that the peak in 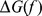 at 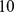 Hz is higher in Fig. 3, compared to that at 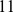 Hz in Fig. 1. Tranfer entropy produced similar results, implying the directionality of coupling as 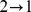.

Fig. 5 and 6 represent the scenario where the effects associated with phase precedence counteract the effects related to the causal relations as implemented in system (15). Specifically, the phase difference 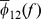 between the two signals at 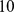 Hz is about 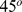, which is interpreted as the phase delay of the driver 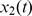 with respect to the response 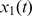. The effects related to the phase shift are relatively strong compared to the inherent causality between 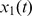 and 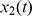. As can be seen from Fig. 5a, the measure of spectral Granger causality switches to negative values. Thus the causal relations are reconstructed as 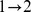, which represents a false-positive case. The measure of transfer entropy is also sensitive to the phase shift, being either positive or negative, depending on the value of the time lag 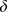. It should be noted that the transfer entropy 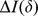 is more resistant to the phase-locking effects, as the mean value 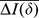 averaged across 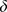 is positive (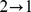).

Notably, the performance of the standard Granger causality was similar to that of the spectral statistic. For no phase shift at 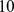 Hz, the mean 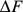 averaged across the realizations was 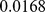, whereas the confidence interval of 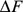 that was based on the corresponding surrogate data and defined by the 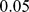
- and 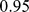-quantiles, was 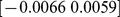. In the case of 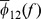 close to 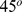 at 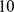 Hz, we had 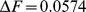 with the confidence interval of 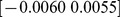 for surrogate data. However, when 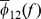 is about 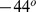, the analysis produced 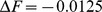, whereas the confidence interval for surrogate data was found to be 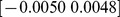. Thus, the standard Granger causal statistic was significantly affected by the differences in phase between the two signals.

#### Aggregated performance

**Figure 7:**
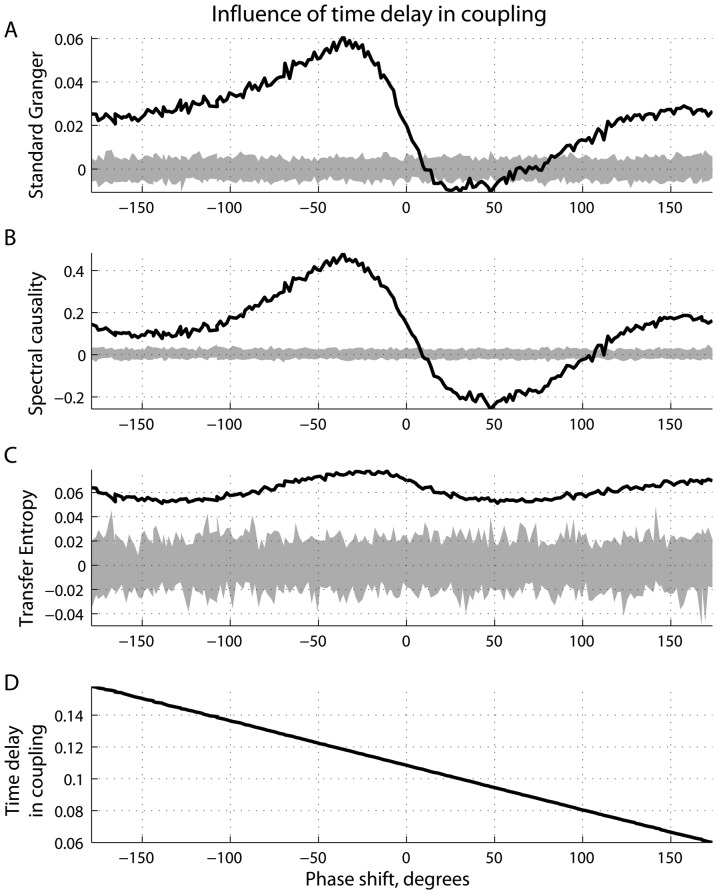
Influence of time delay in coupling. (A) Standard Granger causality; (B) spectral causality; (C) transfer entropy as functions of the observed time diffrence at 10 Hz; and (D) phase difference at 10 Hz as a function of the time delay in coupling, given that the strength of coupling was unchanged.

**Figure 8:**
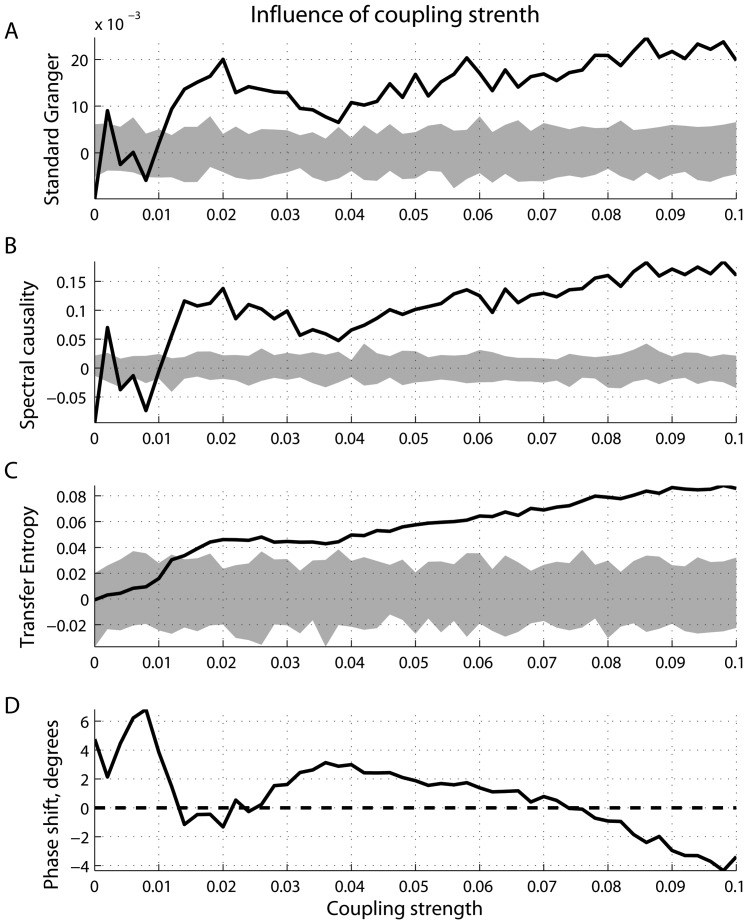
Influence of coupling strength. (A) Standard Granger causality; (B) spectral causality; (C) transfer entropy as functions of phase difference at 10 Hz; and (D) phase difference at 10 Hz as a function of the coupling strength, with the time delay in coupling kept constant.

In the previous section, considering three scenarios, we showed how the effects related to phase advance or phase delay can facilitate or counteract the reconstruction of causal relations. In this section, we focus on the aggregated performance of the causal measures 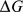 and 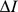, averaged across frequencies 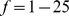 Hz and time lags 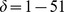, respectively. The measures 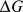 and 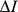 as well as 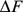 are considered as the functions of the time delay 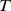 or the strength of coupling 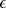. The results of these simulations are reported in Fig. 7 and 8. The solid lines represent the mean values of 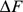, 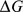 and 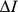, computed for the original data and averaged across realizations. The dark grey area reflects the variability of 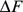, 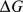, and 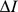, computed for the surrogate data. Specifically, the confidence intervals are defined by the corresponding 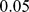
- and 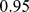-quantiles. The phase difference 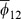 was computed at 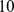 Hz.

Fig. 7 show the results from the simulations wherein the coupling strength was kept constant 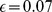, whereas the time delay 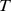 covered the range between 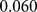 and 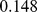. The choice of such values of the parameter 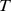 ensures that the phase differences at 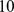 Hz cover the entire period from 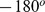 to 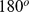, as plotted in Fig. 7c. In turn, the spectral Granger statistic and transfer entropy are given in Fig. 7a and Fig. 7b, respectively, as the functions of 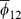 at 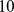 Hz. For all the time lags 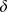, the measure 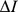 based on information transfer is positive, correctly identifying the causal relations 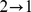. In contrast to 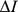, the standard Granger 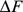 and its spectral version 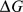 produced false-positive results (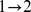) for 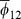 approximately between 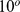 and 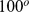. Such values for 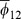 are interpreted as the phase delay of the driver 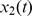 with respect to the response 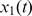.

The phase shift at the frequency when the signals are phase-locked to each other depends not only on the time delay in coupling, but also on the coupling strength 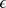. Specifically, Fig. 8 shows the results based on the simulations wherein the time delay 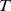 was kept constant 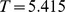, whereas the coupling strength 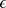 varied from 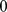 to 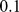. The statistics 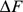, 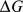 and 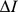 as well as the phase difference 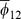 estimated at 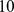 Hz are shown as the functions of 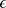. As can be seen from Fig. 8d, 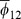 estimated at 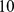 Hz can be either positive (phase delay) or negative (phase lead of driver 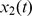 with respect to the response 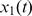).

It is interesting to note that the information-theoretic statistic 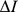 is a monotonic function of 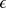 (Fig. 8c). Furthermore, 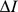 is able to correctly infer the causal relations, producing insignificant values for small coupling strengths, whereas it is indistinguishable from 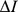 based on the surrogate data. At the same time, both standard Granger and spectral Granger statistics, 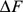 are 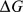, are very sensitive to the phase delay. Specifically, for 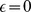 and 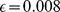 when we observe a phase delay of 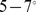 of 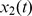 with respect to 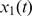 (Fig. 8d), both 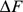 and 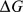 are small, but statistically different from the corresponding populations based on surrogate data (Fig. 8a,b). In other words, the effects related to the phase locking and phase delay are relatively strong compared to the effects associated with modeled causality. If the inherent causal effects are relatively strong (for example, when the coupling strength 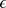 is between 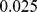 and 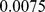, which also corresponds to the phase delay of 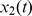 with respect to 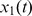 - see Fig. 8c), the standard Granger and spectral Granger statistics correctly identify the directionality of coupling.

### Neurophysiological data

#### Data acquisition

To illustrate the effects similar to those observed in the simulated data, we used an electrocorticography (ECoG) data set provided by the sharing resource NeuroTycho (neurotycho.org). Here we give a brief description of data acquisition and experimental task ( see [40] and [41] for further details). An array of 128 electrodes uniformly covered almost the entire lateral cortex of a monkey brain, from the occipital pole to the temporal and frontal poles. The monkey was sitting in front of a monitor with its head fixed. The data were recorded as a reaction to a visual grating task: a grating pattern which moved in eight directions was presented on a screen. There was no fixation required. Blank and stimulus patterns alternated every 2 sec. In total, there were 160 trials with a monkey watching a black screen, and 20 trials for each of eight directions associated with presenting a visual grating pattern. One cycle of the sinusoid grating pattern covered the distance of 27 mm, moving with a speed of 108 mm/sec, which corresponds to 4 Hz of presentation rate. The distance between the monkey and screen was 490 mm. The ECoG data were sampled at 1 KHz.

In this study, we used the data recorded in reaction to the visual patterns moving horizontally. First, we applied a 50 Hz notch filter. Then, the data were band-pass filtered between 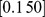
Hz. The time series were normalized to have a zero mean and unit variance. We applied analyses of spectral Granger causality, transfer entropy, and phase locking effects for a pair of electrodes. One electode recorded the signal from the prefrontal polar cortex, close to dorsal prefrontal area 46 (variable 1), whereas the other electrode was localized at the primary visual cortex V1 (variable 2).

#### Analysis

**Figure 9:**
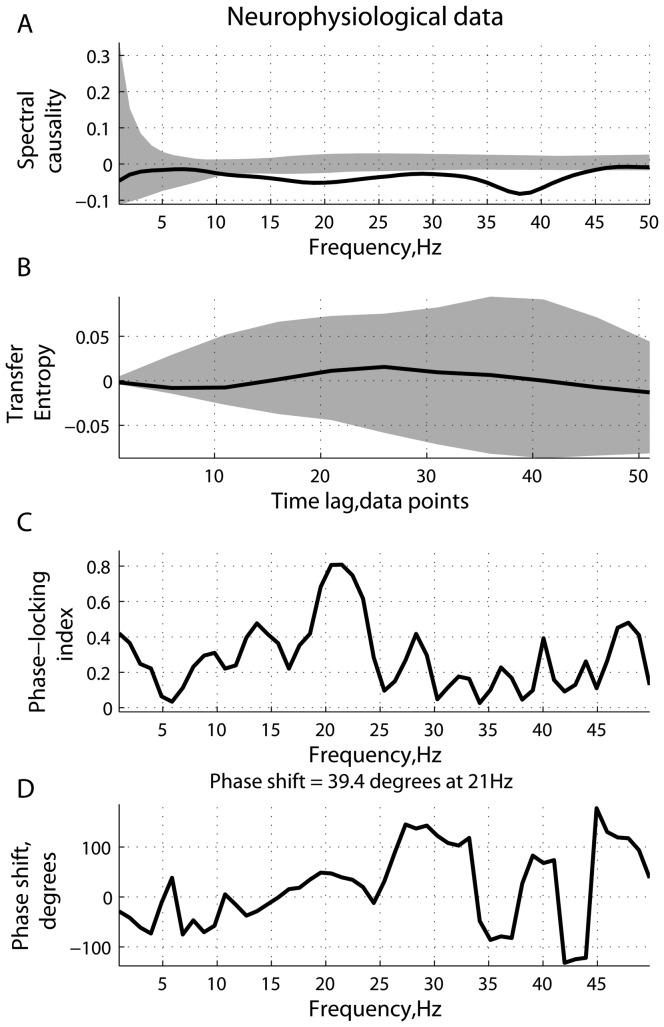
ECoG data: causality and phase synchronization. (A) Estimated spectral causality; (B) transfer entropy; (C) phase-locking index; and (D) phase differences, computed using local field potentials recorded from a pair of ECoG electrodes. Solid lines represent the mean of statistics under investigation, averaged across trials. The shaded area represents the variability (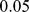
- and 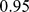-quantiles) of the corresponding statistics based on surrogate data.

**Figure 10:**
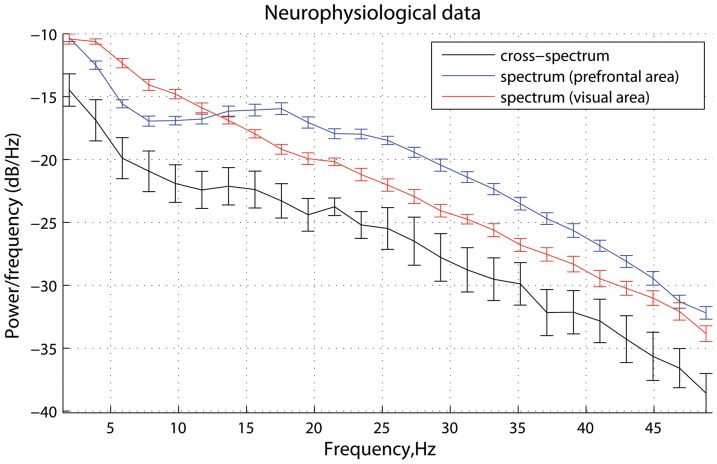
ECoG data: spectral power. Mean spectral density and cross power spectral density of two ECoG channels, averaged across trials. The errorbars represent the standard error computed across trials.

Considering trials as realizations, similar to how it was performed in the case of simulated data, one surrogate time series was created from one observed time series for each trial. Spectral statistics as well as the phase-locking index and phase differences were computed for frequencies 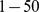 Hz. Transfer entropy was estimated for the time lags 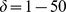. Relations between the spectral Granger causality, transfer entropy, and phase-locking effects are shown in Fig. 9. Spectral and cross-spectral power of the signals are shown in Fig. 10. Similar to Fig. 1, 3, and 5, the solid lines represent the mean of the causal statistics, whereas the shaded area is defined by 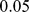
- and 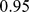-quantiles based on the corresponding surrogate data.

As can be seen from Fig. 9, the signals become strongly phase-locked at 21 Hz, reaching a peak of 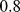 in the phase-locking index. The phase difference at 21 Hz is estimated to be around 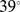, that is, variable 2 (visual cortex) is phase-delayed with respect to variable 1 (prefrontal cortex). Transfer entropy is not sensitive enough to find causality, if any, between the two brain areas. At the same time, the measure of spectral statistic alone indicates the presence of causal effects. The standard Granger statistic reveals similar results, with the mean 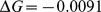 and the confidence interval of 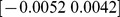. However, this could be explained by the influence of a phase difference at 21 Hz, similar to the results reported in Fig. 5. Thus, caution should be exercised when interpreting the outcome from a causality analysis alone. In the case where the inherent causal coupling, if any, is quite weak, relatively strong phase effects are able to significantly affect a causal statistic, producing spurious results. Possibly, strong phase-locking effects with a noteable phase delay is an indication of the presense of causality, in a general sense, between the two brain areas. However, when it comes to Granger-type causality, an asymmetry in predictive power should not be directly interpreted as “causality”, as it could be reduced to another effect.

## Discussion

The key idea guiding this study is that temporal precedence between harmonic components contributing to the observed signals, which is the basis for Granger causality, does not always imply a corresponding phase advance observed between them. On the contrary, due to ambiguity of phase as a function of time, a phase shift between signals at specific frequencies can be observed either as a phase lead or phase delay. The situation is made worse if the phase shift is not only a function of the time delay in coupling, but also depends on the connection strength. We should expect that in a network with many mutually connected nodes, observed phase differences would be a result of intrinsic combinations of all the connections and time delays in coupling of the network. In turn, the phase difference can have a strong effect on estimation of the driver-response relations, as there exist situations interpreted as the phase delay of the driver with respect to the response.

Two effects can contribute to a causal statistic. The first one is the “true causality” viewed as a result of physical interactions between coupled systems. Statistically, it can be associated with the difference in complexity between the signals at the time scales sensitive to the information exchange [30]. The second effect related to a possible phase difference between signals at specific frequencies can be considered an artifact, which can either intensify or counteract the true causality effects. Depending on the strength of the effects associated with phase difference, the inherent causal effects can be partly neutralized or even totally suppressed. This can be explicitly observed in the scenarios wherein signals become phase-locked to each other at some frequencies, which could have a dominant influence on estimated causal statistics. Note, however, our paper considers the role of phase shifts in the context of non-linear coupled systems, in contrast to the case of linear time-invariant systems. In the scenario of linear transfer functions, the spectrum of the signal is not limited to a single harmonic component but spans several frequencies. In the frequency domain, the slope of the phase (group delay) produces an estimate for the time delay between the signals, which may be used to solve the ambiguity of phase differences at a specific frequency [32], [33]. As can be seen in Fig. 1D, 3D, and 5D, the strategy based on estimating the slope of the phase would be inefficient in the cases we presented.

In this study, we chose a prototypical non-linear model from a wide class of coupled oscillators with time delays in coupling. This was inspired by encouraging results obtained in modeling the resting state network dynamics wherein time delays play a crucial role in the generation of realistic fluctuations in brain signals [6], [7]. We explored how different combinations of the parameters of coupling, the coupling strength, and the time delay in coupling, can lead to either phase lead or phase delay between the driver and the response, which in turn may affect the reconstruction of the directionality of coupling.

The phase differences were considered in the context of phase synchronization and phase-locking, wherein the amount of phase synchrony inherent in two given signals at specific frequencies can be quantified. The directionality of coupling was reconstructed using three measures of Granger causality. First, we used the standard Granger causality, based on autoregressive models that describe both the signals themselves and interactions between them. The second method employs a measure of spectral Granger causality, based on Fourier transform of the autoregressive models. The third approach is a non-linear variant of Granger causality, wherein the asymmetries in the predictive transfer of information between two processes are computed using information-theoretic tools.

In general, we found that all the statistics tested in this study are sensitive to phase differences. However, in our examples, the standard and spectral measures produced statistically significant, but spurious results. The directionality of coupling was identified incorrectly if phase differences between two signals at frequencies where the signals become phase-locked, were approximately in the first quadrant (between 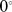 and 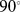), which was interpreted as the phase lead of the response with respect to the driver. On the contrary, the information theoretic measure performed reasonably well in the same situations, correctly reconstructing the underlying relations as specified by the model.

As can be seen from Fig. 7, the standard Granger causality performed slightly better than its spectral version. A key difference between the two tested statistics is that the standard Granger statistic is estimated in the time domain, whereas the spectral measure works in the frequency domain. Thus, the latter explicitly depends on the phase differences between harmonic components of tested signals. On the contrary, causality is ultimately based on interactions between different frequency components. In some sense, inferring the directionality of coupling at a specific frequency can be viewed as an extreme case of filtering the signals with a narrow band-pass filter. The effects of different filtering techniques on the performance of several causality measures have been explored [42]. It was found that, without strong assumptions about the artifacts to be removed, filtering disturbs the information content and leads to missed or spurious results.

Notably, from a general perspective, the spectral causal statistic seems to produce more output, in comparison to the standard Granger causality, in a sense that the directionality of coupling is reconstructed over a range of frequencies of interest. However, there is always a trade off between how much information a statistic can reveal and how reliable that information can be. In this context, a statistic, which works in a time domain and integrates the causal effects across all the frequencies, is able to indicate only the dominant frequency-nonspecific directionality of coupling. On the contrary, the spectral statistics can be more easily mislead in their attempt to capture smaller details in the relations between signals. Even transfer entropy considered as a function of the time lag 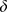 between the past of one signal and the future of another, produced spurious results for some 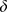.

Nevertheless, transfer entropy performed better than the standard Granger statistic, although both measures work in the time domain. A key difference between these two pipelines is that the transfer entropy was averaged across the time lags 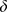 with the aim of decreasing the variability of estimated statistics and increasing the robustness of the results [22]. Specifically, a common practice for computing transfer entropy is to estimate this measure for a range of the time lags 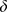, for example, from 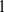 to 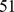 data points, as used in this study. In turn, this time lag 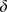 defines the phase difference between the future of a signal and its past. Thus, depending on specific values of 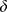, we may observe either phase delay or phase advance of the future of a signal with respect to its past, similar to what we discussed earlier in the context of two signals. If the range of 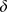 is relatively large to cover the entire period, averaging across 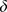 would smooth out the phase effects.

Finally, the findings that an observed measure of causality depended on an intrinsic interplay between inherent causal relations driven by an underlying physical system and an artifact due to differences in phase dynamics, may play a crucial role in testing the significance of connectivity between brain regions in a population. Suppose that we identified two regions of interest or two locations of neural activity in the brain for a group of subjects, and the goal is to estimate the influence one region exerts over the other. One can further assume that the time delay in coupling between two presumably coupled neural systems is the same across subjects, depending approximately on the distance between the regions and propagation speed. Even under such an assumption, the variations in the coupling strength would lead to either the existence of phase delay or phase advance of neural activity in one brain area with respect to another. In the case of weak coupling between the neural systems, the mean causal statistic averaged across subjects might be statistically indistinguishable from zero, even in the best scenario. In the worst case, it could indicate the directionality of coupling opposite to the true relations between given sources of neural activity.
